# Supplementary material for: Folate deficiency among women of reproductive age in Ethiopia: A systematic review and meta-analysis
Source: PLoS One. 2023 May 8;18(5):e0285281. doi: 10.1371/journal.pone.0285281 (PMC10166565; doi:10.1371/journal.pone.0285281)
Supplement: S1 Table — (DOCX) [file pone.0285281.s002.docx]

Supporting Information 2: Risk of bias and quality assessment result of the included 10 studies using the Joanna Briggs Institute (JBI) Critical Appraisal Checklist for Studies Reporting Prevalence Data, 2022

| **S.№** | **Author year** | **Study area** | **Q1** | **Q2** | **Q3** | **Q4** | **Q5** | **Q6** | **Q7** | **Q8** | **Q9** | **Total score (out of 9 points)** | **Decision** |
| --- | --- | --- | --- | --- | --- | --- | --- | --- | --- | --- | --- | --- | --- |
|  | Adela et al., 2018 | Addis Ababa | Y | N | Y | Y | Y | Y | Y | Y | Y | 8/9 | Accepted |
|  | Bekele and Baye, 2019 | Amhara | Y | Y | N | Y | Y | Y | Y | N | Y | 7/9 | Accepted |
|  | Bromage et al, 2021 | National | Y | Y | Y | Y | Y | Y | Y | N | Y | 8/9 | Accepted |
|  | Elema et al., 2018 | Oromia | N | N | N | Y | Y | Y | Y | Y | Y | 6/9 | Accepted |
|  | EPHI, 2016 | National | Y | Y | Y | Y | Y | Y | Y | Y | Y | 9/9 | Accepted |
|  | Gibson et al., 2008 | Sidama | Y | N | N | Y | Y | Y | N | Y | Y | 6/9 | Accepted |
|  | Haidar et al., 2010 | National | Y | Y | N | Y | Y | Y | Y | Y | Y | 8/9 | Accepted |
|  | Kucha et al., 2022 | Addis Ababa | Y | N | N | Y | Y | Y | Y | Y | Y | 7/9 | Accepted |
|  | Mebratu and Baye, 2016 | Oromia | Y | N | N | Y | Y | Y | Y | Y | Y | 7/9 | Accepted |
|  | Yusuf et al., 2021 | Oromia | Y | Y | Y | Y | Y | Y | Y | Y | Y | 9/9 | Accepted |
|  | Number of studies that addressed each item (out of 10) | | 9/10 | 5/10 | 4/10 | 10/10 | 10/10 | 10/10 | 9/10 | 8/10 | 10/10 |  |  |

Risk of bias and quality assessment checklist

| **S.no** | **Items** | **Response categories** | | | |
| --- | --- | --- | --- | --- | --- |
|  |  | **Yes (Y)** | **No (N)** | **Unclear (U)** | **Not applicable (NA)** |
|  | Was the sample frame appropriate to address the target population? (Q1) |  |  |  |  |
|  | Were study participants sampled in an appropriate way? (Q2) |  |  |  |  |
|  | Was the sample size adequate? (Q3) |  |  |  |  |
|  | Were the study subjects and the setting described in detail? (Q4) |  |  |  |  |
|  | Was the data analysis conducted with sufficient coverage of the identified sample? (Q5) |  |  |  |  |
|  | Were valid methods used for the identification of the condition? (Q6) |  |  |  |  |
|  | Was the condition measured in a standard, reliable way for all participants? (Q7) |  |  |  |  |
|  | Was there appropriate statistical analysis? (Q8) |  |  |  |  |
|  | Was the response rate adequate, and if not, was the low response rate managed appropriately? (Q9) |  |  |  |  |

Source: Munn, Z., Moola, S., Lisy, K., Riitano, D., and Tufanaru, C. Methodological guidance for systematic reviews of observational epidemiological studies reporting prevalence and cumulative incidence data. Int J Evid Based Healthc. 2015; 13(3): 147-53. DOI: 10.1097/xeb.0000000000000054.
