# Supplementary material for: Folate deficiency among women of reproductive age in Ethiopia: A systematic review and meta-analysis
Source: PLoS One. 2023 May 8;18(5):e0285281. doi: 10.1371/journal.pone.0285281 (PMC10166565; doi:10.1371/journal.pone.0285281)
Supplement: S1 Text — (DOCX) [file pone.0285281.s004.docx]

**Supplementary Information 1: Full search strategy of studies**

1. **MEDLINE (PubMed): Searched from 1990 up to and including 31 May 2022**

| **Search statement** | **Search terms** | **Search result** |
| --- | --- | --- |
| 1 | (("folate*"[Title/Abstract] OR "folic acid"[Title/Abstract] OR "micronutrient"[Title/Abstract]) AND ("deficien*"[Title/Abstract] OR "status"[Title/Abstract] OR "level"[Title/Abstract])) | 72 |
| 2 | prevalen*[Title/Abstract] OR magnitude[Title/Abstract] |  |
| 3 | Ethiopia*[Title/Abstract] |  |
| 4 | #1 AND #2 AND #3 |  |

1. **Embase (Ovid): Searched from 1990 up to and including 31 May 2022**

| **Search statement** | **Search terms** | **Search results** |
| --- | --- | --- |
| 1 | ('folate*':ti OR 'folic acid':ti OR 'micronutrient':ti) AND ('deficien*':ti OR 'status':ti OR 'level':ti) | 67 |
| 2 | ('folate*':ab OR 'folic acid':ab OR 'micronutrient':ab) AND ('deficien*':ab OR 'status':ab OR 'level':ab) |  |
| 3 | #1 OR #2 |  |
| 4 | 'prevalen*':ti OR 'magnitude':ti |  |
| 5 | 'prevalen*':ab OR 'magnitude’:ab |  |
| 6 | #4 OR #5 |  |
| 7 | 'Ethiopia*':ti |  |
| 8 | 'Ethiopia*':ab |  |
| 9 | #7 OR #8 |  |
| 10 | #3 AND #6 AND #9 |  |

1. **CINAHL (EBSCOhost): Searched from 1999 up to and including 31 May 2022**

| **Search statement** | **Search terms** | **Search results** |
| --- | --- | --- |
| 1 | (MH "Folic Acid Deficiency") OR (MH "Folic Acid+") OR (MH "folate") | 421 |
| 2 | ((AB folate OR AB folic acid OR AB micronutrient AND AB deficiency OR AB status OR AB level)) |  |
| 3 | S1 OR S2 |  |
| 4 | (TI prevalence OR TI magnitude) |  |
| 5 | (AB prevalence OR AB magnitude) |  |
| 6 | S4 OR S5 |  |
| 7 | (TI Ethiopia) |  |
| 8 | (AB Ethiopia) |  |
| 9 | S7 OR S8 |  |
| 10 | S3 AND S6 AND S9 |  |

1. **Google scholar: Searched from 1972 up to and including 31 May 2022**

| **Search statement** | **Search terms** | **Search results** |
| --- | --- | --- |
| 1 | prevalence OR magnitude OR status OR level AND "folate OR folic acid OR micronutrient AND deficiency" AND Ethiopia | 671 |

1. **AJOL (African Journals Online): Searched from 2018 up to and including 31 May 2022**

| **Search statement** | **Search terms** | **Search results** |
| --- | --- | --- |
| 1 | ("prevalence" OR "magnitude" OR "status" OR "level") AND ("folate" OR "folic acid" OR "micronutrient") AND ("deficiency") AND ("Ethiopia") | 329 |

1. **Global Health Data Exchange (GHDx): Searched from 1996 up to and including 31 May 2022**

| **Search statement** | **Search terms** | **Search results** |
| --- | --- | --- |
| 1 | ("prevalence" OR "magnitude" OR "status" OR "level") AND ("folate" OR "folic acid" OR "micronutrient") AND ("deficiency") AND ("Ethiopia") | 4 |

1. **Institutional Repositories of Universities (including Addis Ababa University, Jimma University, Hawassa University, Haramaya University, Arbaminch University, University of Gondar, Bahir Dar University and Mekelle University) and research centers (including Ethiopian Public Health Institute (EPHI), Ethiopian Health and Nutrition Research Institute (EHNRI) and Ethiopian Nutrition Institute (ENI)): Searched from 2002 up to and including 31 May 2022**

| **Search statement** | **Search terms** | **Search results** |
| --- | --- | --- |
| 1 | ("prevalence" OR "magnitude" OR "status" OR "level") AND ("folate" OR "folic acid" OR "micronutrient") AND ("deficiency") AND ("Ethiopia") | 28 |

1. **The citations and lists of references of key articles were looked carefully to collect more articles and increase the searching coverage; with search result = 8**
2. **The Vitamin and Mineral Nutrition Information System (VMNIS) of the World Health Organization (WHO) was also searched, but no result was found.**

**Total search result = 1600 Studies**
