# Supplementary material for: Folate deficiency among women of reproductive age in Ethiopia: A systematic review and meta-analysis
Source: PLoS One. 2023 May 8;18(5):e0285281. doi: 10.1371/journal.pone.0285281 (PMC10166565; doi:10.1371/journal.pone.0285281)
Supplement: S2 Text — (DOCX) [file pone.0285281.s005.docx]

**Folate deficiency among women of reproductive age in Ethiopia: a systematic review and meta-analysis**

Berhe Gebremichael^1^, Hirbo Shore Roba^1, 2^, Alemeshet Getachew^1^, Dejene Tesfaye^3^, Haftu Asmerom^4^

^1^School of Public Health, College of Health and Medical Sciences, Haramaya University, Harar, Ethiopia

^2^School of Health and Medical Sciences, Faculty of Health, Engineering and Sciences, University of Southern Queensland, Queensland, Australia

^3^School of Nursing and Midwifery, College of Health and Medical Sciences, Haramaya University, Harar, Ethiopia

^4^School of Medical Laboratory Science, College of Health and Medical Sciences, Haramaya University, Harar, Ethiopia

Corresponding author: Berhe Gebremichael

Email: [berhegere09@gmail.com](mailto:berhegere09@gmail.com) (BG)

**Abstract**

**Background:** Folate deficiency (FD) can cause adverse health outcomes of public health significance. Although FD is a significant micronutrient deficiency in Ethiopia, concrete evidence is limited. Therefore, this systematic review and meta-analysis were designed to estimate the pooled prevalence of FD among women of reproductive age (WRA).

**Methods: A** systematic literature search was performed using MEDLINE, Embase, CINAHL, Google Scholar, African Journals Online (AJOL), The Vitamin and Mineral Nutrition Information System (VMNIS) of the World Health Organization (WHO), Global Health Data Exchange (GHDx), and institutional repositories of major universities and research centers. Additionally, we scanned the reference lists of relevant articles. Two authors independently selected the studies, extracted the data, and the study risk of bias. Heterogeneity was assessed using the *I^2^* statistic. We used a random-effects model to estimate the pooled mean serum/plasma folate and the pooled prevalence of FD. Begg's and Egger's tests were used to check publication bias.

**Results:** Ten studies—nine cross-sectional and one case-control—with a total of 5,623 WRA were included in the systematic review and meta-analysis. Four (WRA=1,619) and eight (WRA=5,196) cross-sectional studies were used to estimate the pooled mean serum/plasma folate and prevalence of FD, respectively. The pooled mean serum/plasma folate concentration estimate was 7.14 ng/ml (95% CI: 5.73, 8.54), and the pooled prevalence of FD was estimated to be 20.80% (95% CI: 11.29, 32.27). In addition, the meta-regression analysis showed that the sampling technique was significantly associated with mean serum/plasma folate concentration.

**Conclusions:** FD is a significant public health issue among WRA in Ethiopia. Therefore, the public health strategies of the country should focus on promoting the consumption of folate-rich foods, strengthening the coverage of folic acid supplementation and its adherence, and swift translation of the mandatory folic acid fortification into action.

**Systematic review registration:** PROSPERO 2022—CRD42022306266

**Keywords:** Ethiopia, folate, meta-analysis, systematic review, women of reproductive age

**Introduction**

Folate is a naturally occurring essential vitamin, mainly found in green leafy vegetables and legumes ([1](#_ENREF_1)). It is necessary for deoxyribonucleic acid (DNA) replication and normal cell formation and growth ([2](#_ENREF_2)). Therefore, folate deficiency (FD) reduces thymidylic acid and increases homocysteine in the body, resulting in several health risks ([3](#_ENREF_3)).

Folate deficiency is caused primarily by inadequate dietary intake ([4](#_ENREF_4), [5](#_ENREF_5)) and partly by medical and physiologic conditions that increase the need or excretion of folate ([5-8](#_ENREF_5)). Different study findings show that iron deficiency anemia ([4](#_ENREF_4), [9](#_ENREF_9)), knowledge of folate-rich foods ([4](#_ENREF_4), [10](#_ENREF_10)), and folic acid supplementation ([11](#_ENREF_11)) are significantly associated with FD. Folate intake is suboptimal in the diets of many WRA and exacerbated by overcooking foods and poor bioavailability ([8](#_ENREF_8)), estimated to be from 50% to 82% ([12](#_ENREF_12), [13](#_ENREF_13)). In several developed countries, fortifying grains with folic acid has increased folate intake ([14](#_ENREF_14)). However, in Ethiopia, the unavailability of fortified foods further increases the risk of folate insufficiency, particularly for vulnerable populations such as WRA ([4](#_ENREF_4), [15](#_ENREF_15)).

A definition for what constitutes a public health problem for folate deficiency is not well established ([16](#_ENREF_16)) due to limited relevant population-based data ([17](#_ENREF_17)). However, a 5% and above prevalence generally represents a public health problem ([16-19](#_ENREF_16)). Therefore, folate deficiency is considered a severe public health issue, especially among disadvantaged groups in developing countries, including Ethiopia ([20](#_ENREF_20), [21](#_ENREF_21)); some of the most affected groups by FD include women of reproductive age (WRA) ([5](#_ENREF_5), [7](#_ENREF_7), [17](#_ENREF_17)).

According to a global systematic review based on the available evidence of folate status in WRA, the prevalence of FD was greater than 20% in many low-income countries, which is far above the general threshold for public health concerns. However, in high-income countries, the prevalence was less than 5%. In this review, the prevalence of folate insufficiency was more than 40% in most countries ([22](#_ENREF_22)). Among different African countries, the prevalence of FD in pregnant women fluctuated from 0.8% in Kenya to 86.1% in Côte d'Ivoire ([9](#_ENREF_9), [23-26](#_ENREF_23)). Similarly, different study findings in Ethiopia show the prevalence of WRA ranging from 1.9% to 46% ([4](#_ENREF_4), [10](#_ENREF_10), [27-32](#_ENREF_27)). However, in most of these studies, the prevalence of FD was above 5%—indicating its public health significance in the country.

The consequences of folate deficiency among WRA include megaloblastic anemia ([8](#_ENREF_8), [33-35](#_ENREF_33)) and neural tube defects (NTDs) ([36-39](#_ENREF_36)). Maternal anemia is highly prevalent in Ethiopia ([40](#_ENREF_40)); however, reports showing folate deficiency as the etiology of anemia are unavailable. Folate deficiency is a significant risk factor for neural tube defects (NTDs), which affects more than 300,000 babies worldwide and 65 per 10,000 births in Ethiopia ([36-39](#_ENREF_36)). As countries progress in reducing child mortality from infectious diseases, congenital disabilities become a more significant cause of under‐five mortality in many countries ([41](#_ENREF_41)). Other consequences of FD include abortion ([33-35](#_ENREF_33)), preterm birth ([33](#_ENREF_33)), and hyperhomocysteinemia, which is a risk factor for metabolic and cardiovascular diseases ([3](#_ENREF_3), [34](#_ENREF_34), [35](#_ENREF_35), [42](#_ENREF_42)).

Folate deficiency among WRA is one of the significant public health issues in Ethiopia ([43](#_ENREF_43)). Therefore, to address this problem, the Ethiopian Government adopted the global targeted iron and folic acid supplementation for pregnant women during their antenatal care visits to reduce the prevalence of anemia in WRA and children under five ([44](#_ENREF_44)). Additionally, in 2022, the country endorsed the mandatory fortification of edible oil and wheat flour with folic acid, an effective intervention strategy to overcome the burden of FD ([45](#_ENREF_45)). However, to our knowledge, no systematic review and meta-analysis study has addressed FD among WRA in Ethiopia to provide high-level evidence for policymakers to track the progress, evaluate the impact of existing programs and design further context-specific interventions. Therefore, this systematic review and meta-analysis were designed to address the gap and estimate the exact prevalence of FD among WRA in Ethiopia.

**Methods and materials**

**Registration**

This systematic review and meta-analysis were performed, according to the protocol registered in the International Prospective Register of Systematic Reviews (PROSPERO) on 22 February 2022, with registration ID: CRD42022306266.

**Search strategy**

Studies were identified by searching electronic databases, institutional and organizational repositories, and websites. Additionally, the reference lists of key articles were examined to retrieve additional related studies. From the electronic databases, we searched MEDLINE (via PubMed), Embase (via Ovid), CINAHL (via EBSCOhost), African Journals Online (AJOL), and Google Scholar. The Vitamin and Mineral Nutrition Information System (VMNIS) of the World Health Organization (WHO), Global Health Data Exchange (GHDx), institutional repositories of major universities (including Addis Ababa University, Jimma University, Hawassa University, Haramaya University, Arbaminch University, University of Gondar, Bahir Dar University, and Mekelle University), and the institutional repositories of research centers (including the Ethiopian Public Health Institute (EPHI), Ethiopian Health and Nutrition Research Institute (EHNRI) and Ethiopian Nutrition Institute (ENI)) were searched for reports and unpublished articles.

The search was first conducted on 28 February 2022, and updated on 31 May 2022. Two authors (BG and HSR) performed the search activities independently. The following search terms were used to find all relevant studies in the databases and other sources: "prevalence", "magnitude", "status", "level", "folate", "folic acid", "micronutrient", "deficiency" and "Ethiopia". The search terms were used separately and in combination using the Boolean operators 'OR' and 'AND' (**Search Strategy in S1 Text**). The PRISMA guideline for systematic review ([46](#_ENREF_46)) was used to report the search results.

**Eligibility criteria**

The studies which fulfilled the following criteria were included: all observational study designs (cross-sectional, case-control, and cohort studies) which reported mean or median serum or plasma folate, or prevalence of folate deficiency, published in the English language from 2004 to 2022, full-text articles, conducted among WRA (15-49 years old) in Ethiopia, studies with a response rate greater than 80%, reporting quality assurance methods, and quality assessment score better than 50%. For any studies from the same survey, we selected and included the study that reported the desired outcomes and the extracted variables clearly. Studies were excluded if they were found to have a poor-quality score as per the stated criteria, were review articles, qualitative studies, abstracts, or failed to determine the desired outcomes (i.e., mean or median serum or plasma folate or folate deficiency).

The outcome variable was 'folate deficiency' and was defined according to the WHO recommendation as follows: (1) serum or plasma folate <3 ng/mL or red blood cells (RBC) folate <100 ng/mL (using macrocytic anaemia as a haematological indicator); (2) serum or plasma folate <4 ng/mL or RBC folate <151 ng/mL (using homocysteine concentration as a metabolic indicator). Folate insufficiency (level of folate below which is a risk for NTD) was defined as RBC folate < 400 ng/mL ([47](#_ENREF_47)).

**Study risk of bias and quality assessment**

We assessed the risk of bias and quality of the included studies using the Joanna Briggs Institute (JBI) Critical Appraisal Checklist for Studies Reporting Prevalence Data ([48](#_ENREF_48)). The checklist contains nine items: (1) appropriateness of the sample frame to address the target population, (2) appropriateness of sampling procedure/technique, (3) adequacy of sample size, (4) detail description of study subjects and setting, (5) sufficiency of data analysis coverage of the identified sample, (6) validity of methods used for the identification of the condition, (7) measurement of the condition in a standard and reliable way for all WRA, (8) appropriateness of statistical analysis, and (9) adequacy of response rate and appropriateness of management of low response rate.

Two review authors (BG and HSR) independently applied the checklist to assess the risk of bias in each included study. If there were any discrepancies between the two reviewers, first, the two author discussed the issue trying to reach a consensus, with a third review author (AG, DT or HA) acting as an arbiter, if necessary. Finally, studies with a quality assessment score greater than 50% were included in the systematic review; fortunately, all the assessed studies scored above 60% (**Table in S1 Table**).

**Data collection (extraction) process**

A structured data extraction format, prepared using Microsoft Excel 2010, was used to extract all the necessary data. Two reviewers (BG and HSR) independently extracted the data from the eligible studies. Disagreements between the two authors were resolved through discussions and consultations with a third review author (AG, DT or HA). For each included study, we extracted the following study characteristics: authors’ names and publication date, publication year, study setting, study scale/level, study population, sample size, sampling technique, response rate, mean age of WRA, fasting status of WRA during blood sample taking, laboratory assay methods and biomarkers used, cut-off-point used, mean/median serum and/or RBC folate concentration, prevalence of folate deficiency. and associated factors of folate deficiency.

**Data synthesis and analysis**

The meta-analyses were performed using STATA version 16.0 statistical software package. A random-effects model was applied to estimate the pooled mean serum/plasma folate level and the pooled prevalence of FD in WRA ([49](#_ENREF_49)). We used the *metan* and *metaprop* STATA commands to estimate the pooled mean serum/plasma folate and the pooled prevalence of FD, respectively. The standard error for mean serum/plasma folate was generated using the following formula: $se=\frac{\mathrm{SD}}{\sqrt{n}}$ ([50](#_ENREF_50)), where se = standard error, SD = Standard Deviation and n = sample size. We generated and used the standard error instead of standard deviation to give more weight to studies with precise estimates and to include the role of sample size in the estimation.

Forest plots were used to present the results of pooled estimates with a 95% confidence interval (CI). The *I*^2^ statistic was used to test heterogeneity among studies. To explore the sources of heterogeniety, subgroup analysis was done for the mean serum/plasma folate level by survey year and sampling technique. Additionally, subgroup analysis was done for the prevalence of FD by population group and mean age of WRA. Begg's rank correlation test and Egger's linear regression test were used to check for the publication bias. Leave-one-out sensitivity analysis was performed to show how each individual study affects the overall estimate of the rest of the study. Finally, meta-regression analyses were carried out to identify parameters associated with FD.

**Results**

**Study selection process**

Initially, 1600 studies were identified from the electronic searches. Of these, 1560 were published articles, while 40 were thesis and organizational reports. After removing 97 duplicates, 1503 records were screened by title and abstract, and 1467 studies were excluded. Next, 36 records were further assessed for risk of bias and eligibility, and 26 studies were excluded (23 did not meet the eligibility criteria, and three had duplicated content). Finally, ten studies were included in the systematic review and meta-analysis (**Figure 1**).

**Figure 1: PRISMA flow diagram showing the selection process of studies for the systematic review and meta-analysis on folate deficiency among women of reproductive age in Ethiopia, 2022**

**Characteristics of included studies**

This systematic review and meta-analysis included 10 studies ([10](#_ENREF_10), [27-32](#_ENREF_27), [51-53](#_ENREF_51)) with a total of 5,623 WRA, which were conducted between 2004 and 2022. Among the studies, nine ([10](#_ENREF_10), [27-32](#_ENREF_27), [51](#_ENREF_51), [53](#_ENREF_53)) were cross-sectional studies, and one was a case-control ([52](#_ENREF_52)). Regarding the study setting, six of the studies ([27](#_ENREF_27), [28](#_ENREF_28), [30](#_ENREF_30), [31](#_ENREF_31), [51](#_ENREF_51), [53](#_ENREF_53)) were done at the community level, while the other four ([10](#_ENREF_10), [29](#_ENREF_29), [32](#_ENREF_32), [52](#_ENREF_52)) were institutional-based. Three studies ([28](#_ENREF_28), [30](#_ENREF_30), [51](#_ENREF_51)) were conducted at a national level, whereas the other seven were conducted at the sub-national/local level—three in Oromia Region ([29](#_ENREF_29), [32](#_ENREF_32), [53](#_ENREF_53)), one in Amhara Region ([31](#_ENREF_31)), one in Sidama Region ([27](#_ENREF_27)), and two in Addis Ababa City Administration ([10](#_ENREF_10), [52](#_ENREF_52)).

Six studies ([10](#_ENREF_10), [27](#_ENREF_27), [29](#_ENREF_29), [32](#_ENREF_32), [52](#_ENREF_52), [53](#_ENREF_53)) were conducted among pregnant women, and two were ([28](#_ENREF_28), [30](#_ENREF_30)) done among non-pregnant/non-lactating women. However, two studies ([31](#_ENREF_31), [51](#_ENREF_51)) were performed generally in WRA without specifying the physiological status of the women. Five studies ([28](#_ENREF_28), [30](#_ENREF_30), [31](#_ENREF_31), [51](#_ENREF_51), [53](#_ENREF_53)) applied probability sampling techniques, while the other five ([10](#_ENREF_10), [27](#_ENREF_27), [29](#_ENREF_29), [32](#_ENREF_32), [52](#_ENREF_52)) used non-probability sampling. The sample size of the studies ranged from 99 ([27](#_ENREF_27)) to 1647 ([30](#_ENREF_30)), with response rates ranging from 89.5% ([31](#_ENREF_31)) to 100% ([28](#_ENREF_28), [29](#_ENREF_29), [32](#_ENREF_32), [51](#_ENREF_51), [52](#_ENREF_52)). Nine of the studies ([10](#_ENREF_10), [27](#_ENREF_27), [29-32](#_ENREF_29), [51-53](#_ENREF_51)) reported mean (+ SD) age of the WRA, ranging from 24.5 (+ 5.0) ([32](#_ENREF_32)) to 33.1 (+ 7.2) ([31](#_ENREF_31)) years.

Concerning the laboratory methods, three studies ([10](#_ENREF_10), [51](#_ENREF_51), [52](#_ENREF_52)) used fasting blood samples, one ([27](#_ENREF_27)) used non-fasting blood samples, and six studies ([28-32](#_ENREF_28), [53](#_ENREF_53)) did not report the fasting status. Seven studies ([10](#_ENREF_10), [27](#_ENREF_27), [29](#_ENREF_29), [32](#_ENREF_32), [51-53](#_ENREF_51)) used one of the protein binding assays to assess folate status, two studies ([30](#_ENREF_30), [31](#_ENREF_31)) used microbiological assays, and one study ([28](#_ENREF_28)) did not report the type of assay used. Additionally, eight studies ([27-29](#_ENREF_27), [31](#_ENREF_31), [32](#_ENREF_32), [51-53](#_ENREF_51)) reported serum/plasma folate as a biomarker, one reported RBC folate ([10](#_ENREF_10)), and one study reported both serum and RBC folate ([30](#_ENREF_30)).

Out of those included, six of the cross-sectional studies ([27](#_ENREF_27), [29](#_ENREF_29), [31](#_ENREF_31), [51](#_ENREF_51), [53](#_ENREF_53)) reported mean (+ SD) serum/plasma folate concentration, ranging from 4.5 ng/ml (+ 0.1), from Oromia Region ([53](#_ENREF_53)), to 12.6 ng/ml (SD not reported) in Amhara Region ([31](#_ENREF_31)). The single case-control study ([52](#_ENREF_52)) also reported mean (+ SD) serum/plasma folate concentration for the cases (4.4 ng/ml (+ 1.8)), and controls (8.0 ng/ml (+ 3.0)), separately. On the other hand, one cross-sectional study ([32](#_ENREF_32)) reported median (IQR) serum folate concentration as 4.2 ng/ml (2.3) and the case-control study ([52](#_ENREF_52)) reported 4.8 ng/ml (3.7) for the cases and 8.9 ng/ml (5.3) for the controls. Whereas, one study ([10](#_ENREF_10)) reported median (IQR) RBC folate concentration as 585 ng/ml (IQR not reported), and another one study ([30](#_ENREF_30)) reported both median (IQR) serum and RBC folate concentrations as 5.0 ng/ml (4.5) and 255.5 ng/ml (174), respectively.

Eight of the cross-sectional studies ([27-32](#_ENREF_27), [51](#_ENREF_51), [53](#_ENREF_53)) reported FD, fluctuating from 1.9% ([31](#_ENREF_31)) in Amhara Region to 46.0% ([51](#_ENREF_51)) in a national study. Three of these studies ([29](#_ENREF_29), [31](#_ENREF_31), [51](#_ENREF_51)) also reported possible or marginal FD, varying from 2.6% ([31](#_ENREF_31)) to 21.2% ([51](#_ENREF_51)). On the other hand, the case-control study ([52](#_ENREF_52)) merged the FD and possible FD, and found 57% for the cases and 33.5% for the controls. However, one cross-sectional study ([10](#_ENREF_10)) reported folate insufficiency instead of FD.

From all studies, two ([51](#_ENREF_51), [53](#_ENREF_53)) described four factors associated with FD, while another study ([10](#_ENREF_10)) found one associated with folate insufficiency. Accordingly, poor vegetable and grain intake ([51](#_ENREF_51)), poor knowledge on folate-rich foods ([53](#_ENREF_53)), iron deficiency anemia ([51](#_ENREF_51), [53](#_ENREF_53)), and no iron-folic acid supplementation ([53](#_ENREF_53)) were identified as the significant predictors of FD. Similarly, poor vegetable intake ([10](#_ENREF_10)) was significantly associated with folate insufficiency (**Table 1**).

Table 1: Summary characteristics of the 10 studies included in the systematic review and meta-analysis of folate deficiency among women of reproductive age in Ethiopia, 2022

| **Study** | **Study year** | **Study area** | **Population** | **Sampling** | **Study setting** | **Fasting status** | **Lab assay method** | **Bio-marker** | **Cut-off (in ng/ml)** | **Sample size** | **Response rate** | **Mean age (+SD) in yeas** | **Mean folate (+SD) in ng/ml** | **Median folate (IQR) in ng/ml** | **Marginal FD (%)** | **FD (%)** | **Factors associated with FD** |
| --- | --- | --- | --- | --- | --- | --- | --- | --- | --- | --- | --- | --- | --- | --- | --- | --- | --- |
| Adela et al., 2018 | 2017 | Addis Ababa | Pregnant women | Non-probability | Institution | Fasting | PBA (ECLIA; Roche Elecsys®) | RBC | <400 | 160 | 98.8 | 26.5 (4) | NR | 585 (NR) | NR | 27.5* | Reported^a^ |
| Bekele and Baye, 2019 | 2018 | Amhara | WRA | Probability | Community | NR | MBA (5-methyl-THF calibrator) | Serum | <6.6; <4 | 179 | 89.5 | 33.1 (7.2) | 12.6 (NR) | NR | 2.6 | 1.9 | NR |
| Bromage et al, 2021 | 2019 | National | Non-pregnant/non-lactating | Probability | Community | NR | NR | Serum | <3 | 1604 | 100 | NR | NR | NR | NR | 31.8 | NR |
| Elema et al., 2018 | 2015 | Oromia | Pregnant women | Non-probability | Institution | NR | PBA (ECLIA; Roche Elecsys®) | Serum | <3 | 104 | 100 | 24.6 (5) | 7.6 (3.5) | NR | 10.6 | 17.3 | NR |
| EPHI, 2016 | 2015 | National | Non-pregnant | Probability | Community | NR | MBA (calibrator NR) | RBC | <151 | 1647 | 94.5 | 28.9 (8.8) | NR | 255.5 (174) | NR | 32.0 | NR |
|  |  |  |  |  |  |  |  | Serum | <4 | 1647 | 94.5 | 28.9 (8.8) | NR | 5.0 (4.5) | NR | 17.3 | NR |
| Gibson et al., 2008 | 2004 | Sidama | Pregnant women | Non-probability | Community | Non-fasting | PBA (RIA; DPC Dual CountTM Solid Phase No Boil) | Plasma | <3 | 99 | 94.9 | 27.8 (4.6) | 11.5 (5.6) | NR | NR | 2.1 | NR |
| Haidar et al., 2010 | 2005 | National | WRA | Probability | Community | Fasting | PBA (ELISA; Roche Elecsys®) | Plasma | <6.6; <4 | 970 | 100 | 32.6 (12.5) | 5.6 (3.8) | NR | 21.2 | 46 | Reported^b^ |
| Kucha et al., 2022** | 2020 | Addis Ababa | Pregnant women | Non-probability | Institution | Fasting | PBA (ELISA; Cloud-Clone Corp. Katy, TX, USA) | Serum | <6 | Cases: 100 | 100 | Cases: 26.8 (5.3) | Cases: 4.4 (1.8) | Cases: 4.8 (3.7) | Cases: 57 | | NR |
|  |  |  |  |  |  |  |  |  |  | Controls: 167 | 100 | Controls: 27.2 (4) | Controls: 8 (3) | Controls: 8.9 (5.3) | Controls: 33.5 | | NR |
| Mebratu and Baye, 2016 | 2015 | Oromia | Pregnant women | Non-probability | Institution | NR | PBA (ECLIA; Roche cobas® c501) | Serum | <3 | 147 | 100 | 24.5 (5) | NR | 4.2 (2.3) | NR | 21.8 | NR |
| Yusuf et al., 2021 | 2021 | Oromia | Pregnant women | Probability | Community | NR | PBA (ECLIA; Roche cobas® e411) | Serum | <4 | 446 | 96.8 | 25.7 (5.2) | 4.5 (0.1) | NR | NR | 49.3 | Reported^c^ |

SD: standard deviation

IQR: inter-quartile range

FD: folate deficiency

PBA: protein binding assay

ECLIA: electrochemiluminescence immunoassay

RBC: red blood cell

NR: not reported

WRA: women of reproductive age

MBA: microbiological assay

THF: tetrahydrofolate

EPHI: Ethiopia Public Health Institute

RIA: Radioimmunoassay

ELISA: enzyme-linked immunosorbent assay

^a^poor vegetable intake was reported as associated factor

^b^poor vegetable and grain intake, and iron deficiency anemia were reported as associated factors

^c^poor knowledge on folate rich foods, iron deficiency anemia and iron-folic acid supplementation were reported as associated factors

*This study reported folate insufficiency, not folate deficiency

**This is the only case-control study, and it did not report the possible/marginal FD and FD separately; they were merged

**Risk of bias and quality assessment**

The risk of bias and quality of the studies was assessed by the Joanna Briggs Institute (JBI) Critical Appraisal Checklist for Studies Reporting Prevalence Data ([48](#_ENREF_48)), a 9-item based checklist, indicated in the methods and materials section. The overall quality assessment score, with a potential total of 9 points, ranged from 6 (66.7%) to 9 (100%) points. In all (100%) of the included studies, the study subjects and settings were described in detail; valid methods were used for the identification of the condition; the data analysis was performed with sufficient coverage of the identified samples and response rates were adequate. However, the sampling of WRA was inappropriate in 5 (50%) of the studies and sample size was inadequate in 6 (60%) studies (**Table in S1 Table**).

**Mean serum/plasma folate concentration**

Four cross-sectional studies totaling 1,619 WRA were included in the meta-analysis to estimate the pooled mean serum/plasma folate level. Three of the studies ([27](#_ENREF_27), [29](#_ENREF_29), [51](#_ENREF_51)) were published articles, and one was a master thesis ([53](#_ENREF_53)). Two of the studies were from Oromia Region ([29](#_ENREF_29), [53](#_ENREF_53)), one was from Sidama Region ([27](#_ENREF_27)), and one ([51](#_ENREF_51)) was a national study. Concerning the study setting, three studies ([27](#_ENREF_27), [51](#_ENREF_51), [53](#_ENREF_53)) were community-based whereas one ([29](#_ENREF_29)) was an institution-based study. The studies were conducted in 2004 ([27](#_ENREF_27)), 2005 ([51](#_ENREF_51)), 2015 ([29](#_ENREF_29)) and 2021 ([53](#_ENREF_53)). Three studies ([27](#_ENREF_27), [29](#_ENREF_29), [53](#_ENREF_53)) were conducted in pregnant women, while one ([51](#_ENREF_51)) was in WRA. All four studies used protein binding assay to analyze the serum/plasma folate level. Two studies ([51](#_ENREF_51), [53](#_ENREF_53)) used probability sampling and the other two ([27](#_ENREF_27), [29](#_ENREF_29)) used non-probability sampling. The sample size of the studies ranged from 99 ([27](#_ENREF_27)) to 970 ([51](#_ENREF_51)), with a response rate from 94.5% ([27](#_ENREF_27)) to 100% ([29](#_ENREF_29), [51](#_ENREF_51)). The mean (+ SD) serum/plasma folate concentration varies from 4.5 ng/ml (+ 0.1) ([53](#_ENREF_53)) to 11.5 ng/ml (+ 5.6) ([27](#_ENREF_27)) (**Table 1**).

The meta-analysis revealed that the pooled estimate of the mean serum/plasma folate concentration was 7.14 ng/ml (95% CI: 5.73, 8.54). The analysis revealed considerable between-study heterogeneity (*I²=*99.0%, p=0.000) (**Figure 2**). The Egger’s test detected significant publication bias (p=0.000), however, it was not detected in the Begg's test (p=0.09).

**Figure 2: Forest plot of mean serum/plasma folate level among women of reproductive age in Ethiopia, 2022**

**Prevalence of folate deficiency**

The meta-analysis included eight cross-sectional studies conducted from 2004 to 2021, with a total of 5196 WRA, to estimate the pooled prevalence of FD. Four of the studies ([27-29](#_ENREF_27), [51](#_ENREF_51)) were published articles, three ([31](#_ENREF_31), [32](#_ENREF_32), [53](#_ENREF_53)) were master theses, and one ([30](#_ENREF_30)) was an organization survey report. Six of the studies ([27](#_ENREF_27), [28](#_ENREF_28), [30](#_ENREF_30), [31](#_ENREF_31), [51](#_ENREF_51), [53](#_ENREF_53)) were community-based and two ([29](#_ENREF_29), [32](#_ENREF_32)) were institutional-based. Of the eight studies, three ([28](#_ENREF_28), [30](#_ENREF_30), [51](#_ENREF_51)) were done at national level, while five were conducted at the sub-national/local level—three studies were from Oromia Region ([29](#_ENREF_29), [32](#_ENREF_32), [53](#_ENREF_53)), one from Amhara Region ([31](#_ENREF_31)), and one from Sidama Region ([27](#_ENREF_27)). Pregnant women were the target group in four studies ([27](#_ENREF_27), [29](#_ENREF_29), [32](#_ENREF_32), [53](#_ENREF_53)), and non-pregnant/non-lactating women were the targets of two studies ([28](#_ENREF_28), [30](#_ENREF_30)). Nevertheless, two studies ([31](#_ENREF_31), [51](#_ENREF_51)) were done generally in WRA without specifying the physiological status of the women. Five studies ([27](#_ENREF_27), [29](#_ENREF_29), [32](#_ENREF_32), [51](#_ENREF_51), [53](#_ENREF_53)) used protein binding assays, two ([30](#_ENREF_30), [31](#_ENREF_31)) used microbiological assays, and one ([28](#_ENREF_28)) did not report the type of laboratory assay. From the studies, five ([28](#_ENREF_28), [30](#_ENREF_30), [31](#_ENREF_31), [51](#_ENREF_51), [53](#_ENREF_53)) applied probability sampling techniques, while three ([27](#_ENREF_27), [29](#_ENREF_29), [32](#_ENREF_32)) used non-probability sampling. The sample size varied from 99 ([27](#_ENREF_27)) to 1647 ([30](#_ENREF_30)), with response rates ranging from 89.5% ([31](#_ENREF_31)) to 100% ([28](#_ENREF_28), [29](#_ENREF_29), [32](#_ENREF_32), [51](#_ENREF_51)). The prevalence of FD was widespread from 1.9% ([31](#_ENREF_31)) in a study reported from Amhara Region to 46.0% ([51](#_ENREF_51)) in a national study (**Table 1**).

In this meta-analysis, the pooled prevalence of FD was estimated to be 20.80% (95% CI: 11.29, 32.27). The meta-analysis indicated marked evidence of heterogeneity among the studies (*I²=*98.71%, p=0.00) (**Figure 3**). No significant publication bias was detected using Egger's (p=0.51) and Begg's (p=0.17) tests.

**Figure 3: Forest plot of the prevalence of folate deficiency among women of reproductive age in Ethiopia, 2022**

**Subgroup analysis for mean serum/plasma folate concentration**

To explore sources of heterogeneity, subgroup analyses were performed for the mean serum/plasma folate level by survey year and sampling technique. Consequently, the level of mean serum/plasma folate was higher among studies from 2015 or earlier (n=3), (8.18 ng/ml, 95% CI: 5.38, 10.98) compared to those conducted after 2015 (n=1) (4.50 ng/ml, 95% CI: 4.49, 4.51). There was considerable between-group heterogeneity (p=0.01) (**Figure 4**).

**Figure 4: Forest plot of subgroup analysis for mean serum/plasma folate level by survey year among women of reproductive age in Ethiopia, 2022**

Likewise, the mean serum/plasma folate was higher among studies which used non-probability sampling techniques (n=2) (9.52 ng/ml, 95% CI: 5.70, 13.34) compared to those that used probability sampling (n=2) (5.04 ng/ml, 95% CI: 3.97, 6.12). Significant heterogeneity was observed between the groups (p=0.03) (**Figure 5**).

**Figure 5: Forest plot of subgroup analysis for mean serum/plasma folate level by sampling technique among women of reproductive age in Ethiopia, 2022**

**Subgroup analysis for the prevalence of folate deficiency**

We also performed subgroup analyses for the prevalence of FD to find out the possible sources of heterogeneity. Subgroup analysis by population/target group indicated that the prevalence of FD was higher among WRA (unspecified/general population group) (n=2), (36.73%, 95% CI: 33.96, 39.55) than the non-pregnant/non-lactating (n=2), (24.08%, 95% CI: 22.63, 25.57) and pregnant women (n=4), (20.01, 95% CI: 3.34, 45.44). The between-groups heterogeneity was significant (p=0.000) (**Figure 6**).

**Figure 6: Forest plot of subgroup analysis for the prevalence of folate deficiency by population group among women of reproductive age in Ethiopia, 2022**

In the same way, the prevalence of FD was higher among women older than 30 (n=2), (36.73%, 95% CI: 33.96, 39.55) compared to those aged 30 years or less (n=5), (19.53%, 95% CI: 7.10, 36.10). Significant between-groups heterogeneity was observed (p=0.045) (**Figure 7**).

**Figure 7: Forest plot of subgroup analysis for the prevalence of folate deficiency by mean age among women of reproductive age in Ethiopia, 2022**

**Sensitivity analysis**

We performed influential sensitivity (leave-one-out) analysis to assess the effect of each individual study on the observed heterogeneity. Accordingly, a meta-analysis was performed on each subset of the study by leaving out exactly one study and the overall point estimate fell within the CI of each subset. This indicates that no individual study significantly affected the overall estimate of the rest of the studies (**Table 2**).

Table 2: Leave-one-out sensitivity analysis showing the influence of each individual study on the overall estimate in the mean serum/plasma folate level (n=4) and the prevalence of folate deficiency (n=8) among women of reproductive age in Ethiopia, 2022

| **Study omitted** | **Pooled mean serum/plasma folate level in ng/ml (95% CI)** | **Pooled prevalence of folate deficiency (95% CI)** |
| --- | --- | --- |
| Bekele and Baye, 2019 | --- | 24.89 (14.93, 36.41) |
| Bromage et al, 2021 | --- | 19.21 (7.67, 34.32) |
| EPHI, 2016 | --- | 21.30 (10.58, 34.49) |
| Elema et al., 2018 | 6.94 (5.46, 8.43) | 21.29 (11.03, 33.77) |
| Gibson et al., 2008 | 5.84 (4.65, 7.03) | 24.49 (14.09, 36.66) |
| Haidar et al., 2010 | 7.83 (4.25, 11.41) | 17.64 (8.61, 28.98) |
| Mebratu and Baye, 20 | --- | 20.65 (10.44, 33.20) |
| Yusuf et al., 2021 | 8.18 (5.38, 10.98) | 17.31 (8.30, 28.71) |
| **Combined (overall)** | **7.14 (5.73, 8.54)** | **20.80 (11.29, 32.27)** |

**Meta-regression**

Meta-regression was calculated for the mean serum/plasma folate level and FD for covariates to explain the possible sources of heterogeneities observed in the studies. Accordingly, sampling technique was found to be the only covariate significantly associated with mean serum/plasma folate. The application of non-probability sampling technique increased the mean serum/plasma folate level by 4.45 ng/ml (95% CI: 0.57, 8.33; p=0.025) compared to probability sampling method. No covariate showed a significant association with FD (**Table 3**).

Table 3: Meta-regression result of covariates to explain heterogeneity observed in the mean serum/plasma folate level (n=4) and folate deficiency (n=8) among women of reproductive age in Ethiopia, 2022

| **Outcome variable** | **Covariates** | **Coefficient (95% CI)** | **Standard Error** | **P-value** |
| --- | --- | --- | --- | --- |
|  | Survey year | | | |
|  | 2015 or before | 3.70 (-3.01, 10.41) | 0.34 | 0.28 |
|  | After 2015 | Reference |  |  |
|  | Scale of study | | | |
|  | National | -2.23 (-10.12, 5.65) | 4.02 | 0.58 |
|  | Sub-national/local | Reference |  |  |
|  | Study setting | | | |
|  | Community-based | -0.43 (-8.90, 8.02) | 4.31 | 0.92 |
|  | Institution-based | Reference |  |  |
|  | Sampling technique | | | |
|  | Non-probability | **4.45 (0.57, 8.33)** | **1.98** | **0.03** |
|  | Probability | Reference |  |  |
|  | Sample size | | | |
|  | <500 | 2.23 (-5.65, 10.12) | 4.02 | 0.58 |
|  | >500 | Reference |  |  |
|  | Mean age | -0.09 (-1.28, 1.10) | 0.61 | 0.89 |
|  | | | | |
| **Folate deficiency** | Survey year | | | |
|  | 2015 or before | -0.07 (-0.34, 0.21) | 0.14 | 0.64 |
|  | After 2015 | Reference |  |  |
|  | Scale of study | | | |
|  | National | 0.12 (-0.14, 0.38) | 0.13 | 0.34 |
|  | Sub-national/local | Reference |  |  |
|  | Study setting | | | |
|  | Community-based | 0.06 (-0.25, 0.38) | 0.16 | 0.70 |
|  | Institution-based | Reference |  |  |
|  | Population group | | | |
|  | Non-pregnant/non-lactating | -0.01 (-0.41, 0.41) | 0.21 | 0.98 |
|  | Pregnant | -0.01 (-0.38, 0.35) | 0.19 | 0.94 |
|  | WRA (unspecified) | Reference |  |  |
|  | Sampling technique | | | |
|  | Non-probability | 0.16 (-0.42, 0.10) | 0.13 | 0.23 |
|  | Probability | Reference |  |  |
|  | Laboratory assay | | | |
|  | Protein binding | 0.19 (-0.11, 0.49) | 0.15 | 0.21 |
|  | Microbiological | Reference |  |  |
|  | Sample size | | | |
|  | <500 | -0.12 (-0.38, 0.14) | 0.13 | 0.34 |
|  | >500 | Reference |  |  |
|  | Mean age | -0.01 (-0.52, 0.04) | 0.02 | 0.85 |

**Discussion**

Given that there is a paucity of evidence on folate status, particularly in low-income countries ([22](#_ENREF_22)), this systematic review and meta-analysis was a step towards estimating the pooled mean serum/plasma folate and prevalence of FD among WRA—a vulnerable population group—in Ethiopia, a low-income country. This will provide an essential step for policymakers and other stakeholders in designing, planning, and implementing future context-specific interventions on folate.

Ten studies were eligible for this review; however, two ([10](#_ENREF_10), [52](#_ENREF_52)) were not considered in the meta-analyses. One of these two studies reported folate insufficiency instead of FD ([10](#_ENREF_10)), and the other reported both possible/marginal FD and FD combined into one ([52](#_ENREF_52)). On the other hand, only two studies reported factors associated with FD. We did not perform a meta-analysis because iron deficiency anemia was the single factor reported in these studies ([51](#_ENREF_51), [53](#_ENREF_53)). Therefore, we calculated pooled mean serum/plasma folate and pooled prevalence of FD from four ([27](#_ENREF_27), [29](#_ENREF_29), [51](#_ENREF_51), [53](#_ENREF_53)) and eight ([27-32](#_ENREF_27), [51](#_ENREF_51), [53](#_ENREF_53)) studies, respectively.

The current meta-analysis revealed that the pooled mean serum/plasma folate estimate was 7.14 ng/ml (95% CI: 5.73, 8.54). It was significantly higher among studies that applied non-probability sampling and those conducted in 2015 or earlier. Besides, the meta-regression analysis showed that the non-probability sampling technique was significantly associated with the mean serum/plasma folate. Although there is no comparative meta-analysis, the mean serum/plasma folate was consistent with study findings from Nepal (5.9 ng/ml) ([54](#_ENREF_54)), Georgia (7.2 ng/ml) ([55](#_ENREF_55)), Iran (8.0 ng/ml) ([56](#_ENREF_56)), Lebanon (8.4 ng/ml) ([57](#_ENREF_57)) and Senegal (8.5 ng/ml) ([24](#_ENREF_24)). However, it was lower than the findings of various studies in different parts of the world, where the mean serum/plasma folate ranged from 8.6 ng/ml in Austria to 16.3 ng/ml in Ecuador ([58-68](#_ENREF_58)). On the other hand, it was slightly higher than one national study included in this meta-analysis (5.6 ng/ml) ([4](#_ENREF_4)). Additionally, it was higher than the findings of several primary studies, where the mean serum/plasma folate ranged from 2.3 ng/ml in Mongolia to 5.7 ng/ml in Turkey ([23](#_ENREF_23), [69-74](#_ENREF_69)).

The discrepancies in the mean serum/plasma folate could be due to the differences in socio-economic status, study scale, population group, study period, blood sample collection and storage method, the type of laboratory assay used, and eating habits. Our meta-analysis focused on one of the low income-countries (i.e., Ethiopia) with limited coverage and adherence to folic acid supplementation. Although mandatory folic acid fortification has been endorsed by the country ([45](#_ENREF_45)), its translation into action has been limited. Contrary to our review, most other studies were primary studies (not meta-analyses) reported from middle- and high-income countries. Additionally, these middle- and high-income countries have better coverage and adherence to folic acid supplementation and a long history of endorsing mandatory folic acid fortification and effective implementation.

The other point is that different studies use different methods and procedures for blood sample collection and storage and laboratory analysis, such as laboratory assays. Folate results show poor comparability across the laboratory methods, sometimes even within the same analytical technique ([75](#_ENREF_75), [76](#_ENREF_76)). A single assay with different calibrators and laboratory set-ups can even produce different results ([22](#_ENREF_22)), making it challenging to compare folate concentrations across surveys.

Differences in dietary habits across communities, including fasting and folic acid supplementations, could also explain the discrepancies, as serum/plasma folate is easily affected by recent intake ([77](#_ENREF_77)). Serum folate concentrations are approximately 10% higher in nonfasting than in fasting persons, complicating sample collection in field studies ([78](#_ENREF_78)).

A significant driver of much of the recent public health attention to the importance of folate has been the link to NTDs ([16](#_ENREF_16)). In light of this, our systematic review and meta-analysis highlighted that the pooled prevalence of FD was 20.80% (95% CI: 11.29, 32.27), which was four times higher than the threshold for a country-wide public health problem (i.e., greater than 5%) ([16-19](#_ENREF_16)). Therefore, FD is clearly a concern in Ethiopia. Furthermore, significant differences were observed in the sub-group analysis by population group and mean age of WRA; meaning the prevalence was higher among WRA (unclassified population group) than the non-pregnant/non-lactating and pregnant women. In addition, it was also higher among women above 30 years. However, no covariate was significantly associated with FD.

The prevalence of FD in this meta-analysis was consistent with one systematic review and meta-analysis, which pooled 45 surveys conducted in 39 countries from 2000 to 2014. Only 11 surveys were from lower economic countries; the pooled FD was > 20% for most countries ([22](#_ENREF_22)). Likewise, it was consistent with a national survey report (17.3%) included in our meta-analysis. However, the prevalence was higher than two study findings reported from India, with an FD prevalence of 3.2% and 3.5% ([61](#_ENREF_61), [74](#_ENREF_74)) and another study from Belize (4.1%) ([9](#_ENREF_9)). On the contrary, the prevalence of FD was lower in our meta-analysis than in one primary study done in Senegal (54.8%) ([79](#_ENREF_79)).

The differences in socio-economic and demographic characteristics, study scale, setting, period and population, laboratory methods, cut-offs, and feeding habits could explain the variations in the prevalence of FD. The three studies ([9](#_ENREF_9), [61](#_ENREF_61), [74](#_ENREF_74)) with inconsistent findings with our meta-analysis were single (not meta-analyses). This could be explained by the fact that a single primary study may not be comparable with a meta-analysis, which results from statistical analysis of several studies. The other reason for the difference could be the differences in the study population. For example, the Indian studies focused only on pregnant women. In most settings, pregnant women have more access to folic acid supplementation than other subgroups of WRA. On the other hand, Belize is an upper middle-income country, which adopted the mandatory folic acid fortification into wheat flour in 1998 ([80](#_ENREF_80)), whereas Ethiopia adopted it in 2022 ([45](#_ENREF_45)). This could be why the prevalence of FD is much lower in Belize than in our meta-analysis. The prevalence of FD in the Senegalese study was far higher than in our meta-analysis. The target group in the Senegalese study was WRA, where 90.8% were not pregnant ([79](#_ENREF_79)). This means most do not have access to folic acid supplementation provided during pregnancy as part of antenatal care.

Most importantly, the differences in FD could be due to variations in laboratory assays, cut-offs, and dietary practices ([22](#_ENREF_22), [77](#_ENREF_77)). Folate is not stable and is found in the body in numerous forms. Therefore, the various assays have a differing affinity to the different active forms of folate ([22](#_ENREF_22)). Furthermore, studies use different cut-off points depending on the type of indicator used to define FD. In our meta-analysis, the included studies used two sets of cut-offs published by the WHO ([47](#_ENREF_47)). Therefore, folate status should be measured using a microbiological assay as recommended by the WHO that is consistent with regard to common reagents and protocols along with assay-matched cut-offs to define FD and insufficiency for appropriate comparisons across studies ([47](#_ENREF_47), [81](#_ENREF_81)).

The systematic review and meta-analysis findings can be used as input for policies and programs and a gateway for further epidemiological and nutritional studies and meta-analyses. However, the systematic review and meta-analysis have some limitations. For example, we could not produce the pooled estimates for folate insufficiency (an indicator of the risk of NTDs) and factors associated with FD or insufficiency due to the scarcity of adequate primary studies. Additionally, we could not estimate the pooled mean or prevalence of FD using RBC folate, which reflects long-term folate status, because few studies used this biomarker. Moreover, using different laboratory assays may have under or overestimated the results.

**Conclusions**

The systematic review and meta-analysis highlighted that the pooled mean serum/plasma folate concentration among WRA was relatively low. Significant differences were observed in mean serum/plasma folate concentration depending on the sampling techniques used by the studies and the study period (survey year). The pooled prevalence of FD was reported in one-fifth of the WRA, indicating its public health significance in Ethiopia. There was a significant difference in the prevalence by population group and mean age of WRA.

Therefore, the Ethiopian health and nutrition policies should design and strengthen public health strategies to address FD among WRA. Since the magnitude of FD varies throughout the country, targeted interventions are required to address the deficiency. Public health messages should be disseminated through different platforms to improve women’s knowledge of folate, its deficiency, prevention strategies, and folate-rich foods. The availability of folate-rich foods should be ensured by improving production, processing, preservation, pricing, and marketing. Additionally, the consumption of folate-rich foods and folic acid supplementation with a particular focus on coverage and adherence should be strengthened. Preconception, folic acid supplementation should also be considered to reduce NTDs. Folic acid fortification is considered one of the most successful public health initiatives to reduce NTDs and has been an effective intervention strategy in many countries. Thus, the government of Ethiopia, in collaboration with partners, should invest more in folic acid fortification. The mandatory folic acid fortification endorsed by the country should be transformed into action.

**Data Availability Statements**

All the relevant data are included in the article/supplementary material. Further inquiries can be directed to the corresponding author.

**Ethics Statement**

Not applicable

**Author Contributions**

BG originally designed the systematic review and meta-analysis. BG, HSR, AG, DT and HA equally contributed to the study searching and selection, quality and risk of bias assessment, data extraction, and analysis. All authors contributed to writing the manuscript and approved the final manuscript for publication.

**Funding**

The authors received no financial support for the systemmatic review and meta-analysis, authorship, and publication of this article.

**Conflict of Interest**

The author(s) declared no potential conflicts of interest with respect to the systemmatic review and meta-analysis, authorship, and publication of this article.

**Abbreviations**

**AJOL**: African Journals Online, **CI**: Confidence Interval, **CINAHL**: Cumulative Index to Nursing and Allied Health Literature, **FD**: Folate Deficiency, **GHDx**: Global Health Data Exchange, **IQR**: Inter-quartile Range, **NTD**: Neural Tube Defect, **RBC**: Red Blood Cell, **SD**: Standard Deviation, **VMNIS**: Vitamin and Mineral Nutrition Information System, **WHO**: World Health Organization, **WRA**: Women of Reproductive Age

**Reference**

1. Gazzali, A.M., Lobry, M., Colombeau, L. *et al.* (2016). Stability of folic acid under several parameters. *Eur J Pharm Sci* **93**, Suppl. 419-30. DOI: 10.1016/j.ejps.2016.08.045.

2. Cordero, A.M., Crider, K.S., Rogers, L.M. *et al.* (2015). Optimal serum and red blood cell folate concentrations in women of reproductive age for prevention of neural tube defects: World Health Organization guidelines. *MMWR Morb Mortal Wkly Rep* **64**, Suppl. 15, 421-3.

3. Green, R. (2011). Indicators for assessing folate and vitamin B-12 status and for monitoring the efficacy of intervention strategies. *Am J Clin Nutr* **94**, Suppl. 2, 666s-72s. DOI: 10.3945/ajcn.110.009613.

4. Haidar, J., Melaku, U., and Pobocik, R.S. (2010). Folate deficiency in women of reproductive age in nine administrative regions of Ethiopia: an emerging public health problem. *South African Journal of Clinical Nutrition* **23**, Suppl. 3, 132-7.

5. Allen, L.H. (2008). Causes of vitamin B12 and folate deficiency. *Food Nutr Bull* **29**, Suppl. 2 Suppl, S20-37. DOI: 10.1177/15648265080292s105.

6. McNulty, H. and Scott, J.M. (2008). Intake and status of folate and related B-vitamins: considerations and challenges in achieving optimal status. *Br J Nutr* **99 Suppl 3**, Suppl. S48-54. DOI: 10.1017/s0007114508006855.

7. Antony, A.C. (2007). In utero physiology: role of folic acid in nutrient delivery and fetal development. *Am J Clin Nutr* **85**, Suppl. 2, 598s-603s. DOI: 10.1093/ajcn/85.2.598S.

8. McNulty, H. (1995). Folate requirements for health in different population groups. *Br J Biomed Sci* **52**, Suppl. 2, 110-9.

9. Abdelrahim, II, Adam, G.K., Mohmmed, A.A. *et al.* (2009). Anaemia, folate and vitamin B12 deficiency among pregnant women in an area of unstable malaria transmission in eastern Sudan. *Trans R Soc Trop Med Hyg* **103**, Suppl. 5, 493-6. DOI: 10.1016/j.trstmh.2008.10.007.

10. Adela, A., Seifu, D., Menon, M. *et al.* (2018). Assessment of RBC-folate level and other determinant factors of neural tube defect among first trimester pregnant women who attends antenatal care in Lideta and T/haimanot health centers, Addis Ababa, Ethiopia. Suppl. Available from: <http://etd.aau.edu.et/handle/123456789/13438>.

11. Handiso, Y.H., Belachew, T., Abuye, C. *et al.* (2021). A community-based randomized controlled trial providing weekly iron-folic acid supplementation increased serum- ferritin, -folate and hemoglobin concentration of adolescent girls in southern Ethiopia. *Sci Rep* **11**, Suppl. 1, 9646. DOI: 10.1038/s41598-021-89115-5.

12. Institute of Medicine. (1998). Dietary Reference Intakes for Thiamin, Riboflavin, Niacin, Vitamin B(6), Folate, Vitamin B(12), Pantothenic Acid, Biotin, and Choline. Suppl. DOI: 10.17226/6015.

13. Winkels, R.M., Brouwer, I.A., Siebelink, E. *et al.* (2007). Bioavailability of food folates is 80% of that of folic acid. *Am J Clin Nutr* **85**, Suppl. 2, 465-73. DOI: 10.1093/ajcn/85.2.465.

14. Cusick, S.E., Mei, Z., Freedman, D.S. *et al.* (2008). Unexplained decline in the prevalence of anemia among US children and women between 1988-1994 and 1999-2002. *Am J Clin Nutr* **88**, Suppl. 6, 1611-7. DOI: 10.3945/ajcn.2008.25926.

15. FMOH. (2016). Guidelines for the Prevention and Control of Micronutrient Deficiencies in Ethiopia. Suppl. Available from: <http://repository.iifphc.org/bitstream/handle/123456789/1027/Micronutrient%20guideline.pdf?sequence=1&isAllowed=y>.

16. Bailey, L.B., Stover, P.J., McNulty, H. *et al.* (2015). Biomarkers of Nutrition for Development-Folate Review. *J Nutr* **145**, Suppl. 7, 1636s-1680s. DOI: 10.3945/jn.114.206599.

17. McLean, E., de Benoist, B., and Allen, L.H. (2008). Review of the magnitude of folate and vitamin B12 deficiencies worldwide. *Food Nutr Bull* **29**, Suppl. 2 Suppl, S38-51. DOI: 10.1177/15648265080292s107.

18. World Health Organization and Organization., F.a.A. (2006). Guidelines on food fortification with micronutrients. Geneva, Switzerland: World Health Organization. Suppl.

19. de Benoist, B. (2008). Conclusions of a WHO technical consultation on folate and vitamin B12 deficiencies. *Food Nutr Bull.* **29**, Suppl. 2, S238-S244.

20. Harika, R., Faber, M., Samuel, F. *et al.* (2017). Micronutrient Status and Dietary Intake of Iron, Vitamin A, Iodine, Folate and Zinc in Women of Reproductive Age and Pregnant Women in Ethiopia, Kenya, Nigeria and South Africa: A Systematic Review of Data from 2005 to 2015. **9**, Suppl. 10, 1096. Available from: <https://www.mdpi.com/2072-6643/9/10/1096>.

21. Nunn, R.L., Kehoe, S.H., Chopra, H. *et al.* (2019). Dietary micronutrient intakes among women of reproductive age in Mumbai slums. *Eur J Clin Nutr* **73**, Suppl. 11, 1536-1545. DOI: 10.1038/s41430-019-0429-6.

22. Rogers, L.M., Cordero, A.M., Pfeiffer, C.M. *et al.* (2018). Global folate status in women of reproductive age: a systematic review with emphasis on methodological issues. *Ann N Y Acad Sci* **1431**, Suppl. 1, 35-57. DOI: 10.1111/nyas.13963.

23. Rohner, F., Northrop-Clewes, C., Tschannen, A.B. *et al.* (2014). Prevalence and public health relevance of micronutrient deficiencies and undernutrition in pre-school children and women of reproductive age in Côte d'Ivoire, West Africa. *Public Health Nutr* **17**, Suppl. 9, 2016-28. DOI: 10.1017/s136898001300222x.

24. Ndiaye, N.F., Idohou-Dossou, N., Diouf, A. *et al.* (2018). Folate Deficiency and Anemia Among Women of Reproductive Age (15-49 Years) in Senegal: Results of a National Cross-Sectional Survey. *Food Nutr Bull* **39**, Suppl. 1, 65-74. DOI: 10.1177/0379572117739063.

25. Ouédraogo, S., Koura, G.K., Accrombessi, M.M. *et al.* (2012). Maternal anemia at first antenatal visit: prevalence and risk factors in a malaria-endemic area in Benin. *Am J Trop Med Hyg* **87**, Suppl. 3, 418-24. DOI: 10.4269/ajtmh.2012.11-0706.

26. Mgamb, E., Gura, Z., Wanzala, P. *et al.* (2017). Folate deficiency and utilization of folic acid fortified flour among pregnant women attending antenatal clinic at Pumwani Maternity Hospital, Kenya, 2015. *Pan Afr Med J* **28**, Suppl. Suppl 1, 8. DOI: 10.11604/pamj.supp.2017.28.1.9296.

27. Gibson, R.S., Abebe, Y., Stabler, S. *et al.* (2008). Zinc, gravida, infection, and iron, but not vitamin B-12 or folate status, predict hemoglobin during pregnancy in Southern Ethiopia. *J Nutr* **138**, Suppl. 3, 581-6. DOI: 10.1093/jn/138.3.581.

28. Bromage, S., Andersen, C.T., Tadesse, A.W. *et al.* (2021). The global diet quality score is associated with higher nutrient adequacy, mid-upper arm circumference, venous hemoglobin, and serum folate among urban and rural Ethiopian adults. *J Nutr* **151**, Suppl. 130s-142s. DOI: 10.1093/jn/nxab264.

29. Elema, T.B., Yimam, K.B., Waka, F.C. *et al.* (2018). Folate and Vitamin B-12 status of anemic pregnant women and association to hemoglobin during antenatal care, 17-37 weeks in Ambo Hospital, Oromia, Ethiopia, a multi-regression analysis of socio-economic and serum folate and Vitamin B-12. *J Nutr Hum Health* **2**, Suppl. 1, 28-34.

30. EPHI. (2016). Ethiopian National Micronutrient Survey Report. Suppl. Available from: <https://www.exemplars.health/-/media/files/egh/resources/stunting/ethiopia/ethiopian-national-micronutrient-survey-report.pdf>.

31. Bekele, H. and Baye, K. (2019). The relationship between dietary folate intakes and serum folate status of reproductive age women in West Gojam, Ethiopia. Suppl.

32. Mebratu, L. and Baye, K. (2016). Identification of risk factors for anemia on third trimester pregnant women West Showa Zone, Ambo, Ethiopia. Suppl. Available from: <http://etd.aau.edu.et/handle/123456789/1527>.

33. Siega-Riz, A.M., Savitz, D.A., Zeisel, S.H. *et al.* (2004). Second trimester folate status and preterm birth. *Am J Obstet Gynecol* **191**, Suppl. 6, 1851-7. DOI: 10.1016/j.ajog.2004.07.076.

34. Rodrigues, H.G., Gubert, M.B., and Santos, L.M. (2015). Folic acid intake by pregnant women from Vale do Jequitinhonha, Brazil, and the contribution of fortified foods. *Arch Latinoam Nutr* **65**, Suppl. 1, 27-35.

35. Arias, L.D., Parra, B.E., Muñoz, A.M. *et al.* (2017). Study Exploring the Effects of Daily Supplementation with 400 μg of Folic Acid on the Nutritional Status of Folate in Women of Reproductive Age. *Birth Defects Res* **109**, Suppl. 8, 564-573. DOI: 10.1002/bdr2.1004.

36. Centeno Tablante, E., Pachón, H., Guetterman, H.M. *et al.* (2019). Fortification of wheat and maize flour with folic acid for population health outcomes. *Cochrane Database Syst Rev* **7**, Suppl. 7, Cd012150. DOI: 10.1002/14651858.CD012150.pub2.

37. Bulloch, R.E., McCowan, L.M.E., Thompson, J.M.D. *et al.* (2019). Plasma folate and its association with folic acid supplementation, socio-demographic and lifestyle factors among New Zealand pregnant women. *Br J Nutr* **122**, Suppl. 8, 910-918. DOI: 10.1017/s0007114519001788.

38. McNulty, H., Ward, M., Hoey, L. *et al.* (2019). Addressing optimal folate and related B-vitamin status through the lifecycle: health impacts and challenges. *Proc Nutr Soc* **78**, Suppl. 3, 449-462. DOI: 10.1017/s0029665119000661.

39. Bitew, Z.W., Worku, T., Alebel, A. *et al.* (2020). Magnitude and Associated Factors of Neural Tube Defects in Ethiopia: A Systematic Review and Meta-Analysis. *Glob Pediatr Health* **7**, Suppl. 2333794x20939423. DOI: 10.1177/2333794x20939423.

40. Kibret, K.T., Chojenta, C., D'Arcy, E. *et al.* (2019). Spatial distribution and determinant factors of anaemia among women of reproductive age in Ethiopia: a multilevel and spatial analysis. *BMJ Open* **9**, Suppl. 4, e027276. DOI: 10.1136/bmjopen-2018-027276.

41. Liu, L., Oza, S., Hogan, D. *et al.* (2016). Global, regional, and national causes of under-5 mortality in 2000-15: an updated systematic analysis with implications for the Sustainable Development Goals. *Lancet* **388**, Suppl. 10063, 3027-3035. DOI: 10.1016/s0140-6736(16)31593-8.

42. Ganji, V. and Kafai, M.R. (2006). Population reference values for plasma total homocysteine concentrations in US adults after the fortification of cereals with folic acid. *Am J Clin Nutr* **84**, Suppl. 5, 989-94. DOI: 10.1093/ajcn/84.5.989.

43. UNICEF, World Bank, World Food Program *et al.* (2016). Ethiopia National Micronutrient Survey Report. Suppl. 1-60. Available from: <https://www.ephi.gov.et>.

44. Federal Ministry of Health of Ethiopia, *National guideline for control and prevention of micronutrient deficiencies*. 2004, FMOH Addis Ababa.

45. The Ethiopian Standard Council. (2022). Endorsement of the mandatory fortification of edible oil and wheat flour with folic acid. Suppl. Available from: <https://www.nutritionintl.org/news/all-news/government-endorses-mandatory-food-fortification-prevent-high-burden-neural-tube-defects-ethiopia>.

46. Page, M.J., McKenzie, J.E., Bossuyt, P.M. *et al.* (2021). The PRISMA 2020 statement: an updated guideline for reporting systematic reviews. *Syst Rev* **10**, Suppl. 1, 89. DOI: 10.1186/s13643-021-01626-4.

47. WHO. (2015). Serum and red blood cell folate concentrations for assessing folate status in populations. Vitamin and Mineral Nutrition Information System. Geneva: World Health Organization. Suppl. Available from: <http://apps.who.int/iris/bitstream/10665/162114/1/WHO_NMH_NHD_EPG_15.01.pdf?ua=1>.

48. Munn, Z., Moola, S., Lisy, K. *et al.* (2015). Methodological guidance for systematic reviews of observational epidemiological studies reporting prevalence and cumulative incidence data. *Int J Evid Based Healthc* **13**, Suppl. 3, 147-53. DOI: 10.1097/xeb.0000000000000054.

49. DerSimonian, R. and Laird, N. (1986). Meta-analysis in clinical trials. *Control Clin Trials* **7**, Suppl. 3, 177-88. DOI: 10.1016/0197-2456(86)90046-2.

50. Altman, D.G. and Bland, J.M. (2005). Standard deviations and standard errors. *BMJ (Clinical research ed.)* **331**, Suppl. 7521, 903-903. DOI: 10.1136/bmj.331.7521.903. Available from: <https://pubmed.ncbi.nlm.nih.gov/16223828>.

51. Haidar, J., Melaku, U., and Pobocik, R.S. (2010). Folate deficiency in women of reproductive age in nine administrative regions of Ethiopia: an emerging public health problem. *S Afr J Clin Nutr* **23**, Suppl. 3, 132-137.

52. Kucha, W., Seifu, D., Tirsit, A. *et al.* (2022). Folate, Vitamin B12, and Homocysteine Levels in Women With Neural Tube Defect-Affected Pregnancy in Addis Ababa, Ethiopia. *Front Nutr* **9**, Suppl. 873900. DOI: 10.3389/fnut.2022.873900.

53. Yusuf, N., Roba, H.S., and Roba, K.T. (2021). Prevalence of folate deficiency and associated factor among pregnant women in Haramaya District, Eastern Ethiopia Suppl.

54. Chandyo, R.K., Ulak, M., Sommerfelt, H. *et al.* (2016). Nutritional Intake and Status of Cobalamin and Folate among Non-Pregnant Women of Reproductive Age in Bhaktapur, Nepal. *Nutrients* **8**, Suppl. 6. DOI: 10.3390/nu8060375.

55. Ministry of Health and Social Affairs (Georgia), National Centre for Disease Control and Public Health, and Georgia., U. (2010). Report of the 2009 Georgia National Nutrition Survey. Vilnius: United Nations Children’s Fund-Georgia. Suppl.

56. Abdollahi, Z., Elmadfa, I., and Djazayery, A. (2011). Efficacy of flour fortification with folic acid in women of childbearing age in Iran. *Ann. Nutr. Metab.* **58**, Suppl. 188-196.

57. Al Khatib, L., Obeid, O., and Sibai, A.M. (2006). Folate deficiency is associated with nutritional anaemia in Lebanese women of childbearing age. *Public Health Nutr.* **9**, Suppl. 921-927.

58. Institut fur Ernahrungswissenschaften Universitat Wien and Bundesministeriums fur Gesundheit. (2012). Osterreichischer ernahrungsbericht 2012 [Austrian nutrition report 2012]. Vienna: Federal Ministry of Health. Suppl.

59. Bates, B., Prentice, A., and Bates, C. (2015). National Diet and Nutrition Survey Rolling Progamme (NDNS RP). Supplementary report: blood folate results for the UK as a whole, Scotland, Northern Ireland (years 1 to 4 combined) and Wales (years 2 to 5 combined). Public Health England, London. Suppl.

60. Chen, K.J., Pan, W.H., and Lin, Y.C. (2011). Status in the Taiwanese population aged 19 years and older from the Nutrition and Health Survey in Taiwan 1993-1996 to 2005-2008. *Asia Pac. J. Clin. Nutr.* **20**, Suppl. 275-282.

61. Saxena, V., Naithani, M., and Singh, R. (2017). Epidemiological determinants of Folate deficiency among pregnant women of district Dehradun. *Clinical Epidemiology and Global Health* **5**, Suppl. 21-27. DOI: <http://dx.doi.org/10.1016/j.cegh.2016.06.003>.

62. Al-Dallal, Z.S. and Moosa, K. (2003). Impact of the national flour fortification program on the prevalence of iron deficiency and anemia among women at reproductive age in the Kingdom of Bahrain (first monitoring study). Ministry of Health, Juffair. Suppl.

63. Ministry of Health (Argentina). (2007). Encuesta Nacional de Nutricion y Salud (ENNyS) 2004-2005. Documento de Resultados [NationalHealth andNutrition Survey (ENNyS) 2004–2005. Results document]. Buenos Aires: Ministry of Health, Federal Health Plan. Suppl.

64. Shamah-Levy, T., Villalpando, S., and Mejia-Rodriguez, F. (2015). Prevalence of iron, folate, and vitamin B12 deficiencies in 20 to 49 years old women: Ensanut 2012. *Salud P´ublica Mex.* **57**, Suppl. 385-393.

65. Schultz, J.T. and Vatucawaqa, P.T. (2012). Impact of iron fortified flour in child bearing age (CBA) women in Fiji, 2010 report. National Food and Nutrition Centre, Suva. Suppl.

66. University of Otago, M.o.H.N.Z. (2011). A focus on nutrition: key findings of the 2008/09 New Zealand Adult Nutrition Survey. Wellington: Ministry of Health. Suppl.

67. Australian Bureau of Statistics. (2013). Australian Health Survey: biomedical results for nutrients, 2011-12. Canberra: Australian Bureau of Statistics. Suppl.

68. Freire, W., Ramirez-Luzuriaga, M., and Belmont, P. (2014). Tomo I: Encuesta Nacional de Salud y Nutrici ´on de la poblaci ´on ecuatoriana de cero a 59 a˜nos. ENSANUT-ECU 2012.Ministry of Public Health/National Institute of Statistics and Census, Quito. Suppl.

69. Bolormaa, N., Byambatogtoch, B., and Bates, J. (2003). Final report of a survey assessing the nutritional consequences of the dzud in Mongolia. Ulaan Baatar: Mongolia Ministry of Health. Suppl.

70. Gil-Prieto, R., Hern´andez, V., and Cano, B. (2009). Plasma homocysteine in adolescents depends on the interaction between methylenetetrahydrofolate reductase genotype, lipids and folate: a seroepidemiological study. *Nutr. Metab.* **5**, Suppl. 39.

71. Ministry of Health and Sanitation (Sierra Leone), UNICEF, Helen Keller International *et al.* (2015). 2013 Sierra Leone Micronutrient Survey. Freetown: Ministry of Health and Sanitation. Suppl.

72. Ministry of Health (Azerbaijan), State Statistical Committee, and UNICEF-Azerbaijan. (2014). AzerbaijanNutrition Survey (AzNS) 2013. Baku: Ministry of Health. Suppl.

73. Oner, N., Vatansever, U., and Karasalihoglu, S. (2006). The prevalence of folic acid deficiency among adolescent girls living in Edirne, Turkey. *J. Adolesc. Health* **38**, Suppl. 599-606.

74. Finkelstein, J.L., Fothergill, A., Johnson, C.B. *et al.* (2021). Anemia and Vitamin B-12 and Folate Status in Women of Reproductive Age in Southern India: Estimating Population-Based Risk of Neural Tube Defects. *Curr Dev Nutr* **5**, Suppl. 5, nzab069. DOI: 10.1093/cdn/nzab069.

75. Gunter, E.W., Bowman, B.A., Caudill, S.P. *et al.* (1996). Results of an international round robin for serum and whole-blood folate. *Clin Chem* **42**, Suppl. 10, 1689-94.

76. Pfeiffer, C., Gunter, E., and Caudill, S. *Comparison of serum and whole blood folate measurements in 12 laboratories: An international study*. in *Clinical Chemistry*. 2001. Amer Assoc Clinical Chemistry 2101 L Street NW, Suite 202, Washington DC.

77. Sobczynska-Malefora, A. and Harrington, D.J. (2018). Laboratory assessment of folate (vitamin B9) status. *J. Clin. Pathol.* **71**, Suppl. 949.

78. Haynes, B.M., Pfeiffer, C.M., Sternberg, M.R. *et al.* (2013). Selected physiologic variables are weakly to moderately associated with 29 biomarkers of diet and nutrition, NHANES 2003-2006. *J Nutr* **143**, Suppl. 6, 1001s-10s. DOI: 10.3945/jn.112.172882.

79. Ndiaye, N.F., Idohou-Dossou, N., Diouf, A. *et al.* (2018). Folate Deficiency and Anemia Among Women of Reproductive Age (15-49 Years) in Senegal: Results of a National Cross-Sectional Survey *Food and Nutrition Bulletin* **39**, Suppl. 1, 65-74. DOI: 10.1177/0379572117739063.

80. World Health Organization. (1998). Global database on the implementation of nutrition action (GINA). Suppl. Available from: <https://extranet.who.int/nutrition/gina/en/node/25831>.

81. World Health Organization. (2015). Guideline: optimal serum and red blood cell folate concentrations in women of reproductive age for prevention of neural tube defects. Geneva: World Health Organization. Suppl.

**Supporting Information**

S1 Dataset: the dataset from which the results of the study were produced (STATA file)

S1 Table: risk of bias and quality assessment result of the included 10 studies using the Joanna Briggs Institute (JBI) Critical Appraisal Checklist for Studies Reporting Prevalence Data, 2022

S2 Table: PRISMA 2020 checklist

S1 Text: full search strategy of studies

S2 Text: a copy of manuscript with track changes (copyedited for language errors)
