# Supplementary material for: Folate deficiency among women of reproductive age in Ethiopia: A systematic review and meta-analysis
Source: PLoS One. 2023 May 8;18(5):e0285281. doi: 10.1371/journal.pone.0285281 (PMC10166565; doi:10.1371/journal.pone.0285281)
Supplement: Attachment — Submitted filename: review.docx [file pone.0285281.s006.docx]

It’s a well-written and interesting study.

**Abstract**

1. Include the full names of the following abbreviated databases, AJOL, VMNIS, GHDx
2. Right the full names of WHO
3. The recommendation should consider planned and implemented innervations aimed to reduce folate deficiency. The interventions you suggested are already within the government's focus. However, there are shortcomings in the coverage, translating fortification policies into actions. The recommendation should be rewritten in light of this.

**Introduction**

1. The introduction should clearly state why folate deficiency has been considered a public health problem, especially among women of reproductive age.
2. Mention what are the currently implemented (iron-folic acid supplementation) and planned interventions (fortification).
3. The last paragraph should be focused on folate deficiency only.
4. You have stated that “However, evidence on the prevalence of FD among WRA are not strong enough to the level policymakers could develop evidence-based intervention strategies in the country except for folic acid supplementation for pregnant women who visit health facilities for antenatal care follow-up.” However, the Ethiopian Standard Council endorsed the mandatory fortification of edible oil and wheat flour on 10th June 2022, with the currently available evidence. This seems to be in contradiction with your claim. In light of this rewrite the above-mentioned section. You can find this information through this link https://www.nutritionintl.org/news/all-news/government-endorses-mandatory-food-fortification-prevent-high-burden-neural-tube-defects-ethiopia

**Method**

**Search strategy**

1. It has been mentioned that Addis Ababa university instructional repositories have been used. This is a great approach to identifying unpublished studies. Makes sure that the thesis or dissertation you have identified has not been published and cross-check for duplication.
2. Other major national universities' institutional repositories and research institution repositories should also be included in the search strategy.

**Eligibility criteria**

3. Mention how studies from the same survey have been handled
4. In line 107 remove “NOT”, since you haven’t used this boolean operator in the search strategy

**Result**

5. in line 175 remove “Id”
6. summarise characteristics of studies and participants in table format
7. correct table 1, has been split into two places
8. Table 1 has been cited on line number 225, however, this paragraph is not related to the findings reported in table 1.
9. Throughout the manuscript replace “participants” with woman of reproductive age (WRA)
10. Table 1 has been cited on line number 277, however, this paragraph is not related to the findings reported in table 1
11. Perform a sub-group analysis based on the laboratory method used to asses folate concentration If adequate studies can be found within the subgroups.
12. Please include types of laboratory methods used to analyse folate concentration as a covariate in the meta-regression.
13. Discuss further the similarity and difference in mean serum folate concentration and folate deficiency with studies from other nations; explain the similarity and difference from the perspective of implemented interventions aimed at reducing folate deficiency.
14. From lines 391 to 393 you have stated that “All the included studies into the meta-analysis were cross-sectional, and temporal relationship could not be established between the covariates and the outcome variables”. Remove these statements as the aim of this systematic review and meta-analysis is not to establish causality or explore folate deficiency determinants.
15. You have mentioned that different laboratory assays are used. Discuss how the difference in laboratory assays used may affect your finding, and comparability of studies.

**Conclusion**

16. Rewrite the concussion making it clear and concise
17. In line 403 replace “women” with women of reproductive age
18. From lines 403-404 you have stated that “Additionally, the pooled prevalence of FD was reported in one-fifth of the women, indicating its public health significance in Ethiopia.”. It is also possible to justify why folate deficiency is public health problem from the perspective of neural tube defect
19. You have recommended multiple interventions targeting folate deficiency among these interventions fortification has been proven to be the most effective one. On your recommendation, more emphasis should be given to fortification interventions.
